# Restoring Psychology's Role in Peptic Ulcer

**DOI:** 10.1111/j.1758-0854.2012.01076.x

**Published:** 2012-07-30

**Authors:** J Bruce Overmier, Robert Murison

**Affiliations:** University of MinnesotaUSA; University of BergenNorway

**Keywords:** control, Helicobacter pylori, helplessness, prediction, stress, ulcer

## Abstract

This paper reviews the history of the transition from the belief that gastrointestinal ulcers are caused primarily by psychological factors to the current state of belief that they are caused primarily by infection and argues that neither is fully accurate. We argue that psychological factors play a significant role as predisposing to vulnerability, modulating of precipitation, and sustaining of gastric ulceration. We review data that challenge the assumption of a simple infectious disease model and adduce recent preclinical data that confirm the predisposing, modulatory, and sustaining roles for psychological factors. We note that others, too, are now challenging the adequacy of the contemporary simple bacterial infection model. We hope to replace the competition between psychology and medicine with cooperation in understanding and treating patients suffering gastric ulceration and ulcer.

## INTRODUCTION

The constructs of physical health and psychological well-being are both complexly determined and interactive. Nowhere is this clearer than with the so-called psychosomatic disorders. The question of the relevance or not of psychological states in any disorder—and particularly ulcer disease—has its roots in Dualism. Descartes' concept of the reflex arc and the postulation of the body as a machine responding to the immediate environment, and the mind as seat of the soul and passions and separate from the body, opened the possibility of animal models of bodily function and gave rise to modern experimental medicine through the researches of Magendie, Muller, Bernard, and Pavlov. However, the setting aside of the study of the mind, presumed explorable only through introspection (as exploited by the British Associationist philosophers from which much of psychology springs), in essence created—and has sustained—medicine and psychology as separate disciplines having different subject matter and histories, and indeed different forms of science. Darwin, whom we celebrated in 2009, was anti-Dualism, and he argued that there is no qualitative separation between animals and man. It is the essence of Darwin's texts that there is continuity of morphology, adaptive capacity, and emotional and mental functions across species, and this underlies our continuing use of animal models to study all human disorders. Although today we reject simple Dualism, it continues to pervade our common language, and is reflected in the continuing distinction between medical and mental illness in healthcare practices.

The importance of this recounting is because a perceived separation between animals and man and between body and mind is still with us today, and it is the bane of modern health psychology. It is this dualism that gives physicians and health psychologists different standing and authorities in hospitals today. It is Dualism to which most people implicitly subscribe when thinking about health and well-being, and it permeates the literature on peptic ulcer.

## SEARCH FOR THE PRIMARY CAUSE OF PEPTIC (GASTRIC AND DUODENAL) ULCER

Peptic ulcer disease (gastric and duodenal ulcers) has long been and is a continuing serious medical problem, with higher mortality for gastric ulcer than for duodenal ulcer. It is a worldwide problem, despite variations in incidence among countries. In the USA, more than 300,000 cases are diagnosed each year and millions have active disease at any one time with a cost of billions of dollars (e.g. Kurata & Haile, 1984; Sonnenberg & Everhart, 1997). Or, in South Korea in 2005, 20 per cent of all adults visiting a university hospital for any reason were reported to suffer peptic ulcer—with over half being gastric (Kim et al., 2009). PubMed carries some 50,000 references on gastric ulcer. People seek lives free from ulcer disease and its symptomology, and medicine and psychology have each sought to understand the cause of ulcers.

Just over 50 years ago psychologist Joseph Brady and colleagues reported their famous researches on the “Executive Monkeys” (Brady, Porter, Conrad, & Mason, 1958) showing that differences in behavioral-experiential manipulations could cause gastric ulceration and even lead to death. That research caught the interest of many in the public and in the scientific world, because it fit with the then popular—but erroneous—conception of ulcers as a disease of Caucasian managers and foremen, who were those most commonly diagnosed with peptic ulcer (e.g. Bermann, 1961; Robinson & Brucer, 1940). Of course, this was not the first time stress and gastric ulcer had been linked. Selye's (1936) classic discoveries of links between physical stress and ulcer were already well known. Selye linked strains from persistent disease, injury, exertion, and “mental pressure” to the stress syndrome and identified gastric ulcer as the prototypic psychosomatic stress disorder (Selye, 1956). Additionally, Wolff and Wolf (1943), in their fascinating studies with patient Tom, had shown that the status of the stomach was directly influenced by the emotional state of the individual. But it was the work of Brady et al. that stimulated a new round of efforts to identify the psychological causes of gastric ulceration, long presumed (see Alexander & French, 1948).

The search for *the* primary cause of gastric ulcers spanned the last half of the 20th century. From the 1950s to the 1970s, psychologists used animal models to search for unitary psychological conditions that might be the direct causal factor. There were the experiments of Brady and his associates that suggested that psychological stress of “responsibility” spanning weeks and months of action to protect self and others from aversive events—the role of executives—was the causal factor for ulcers. Mason and his colleagues similarly emphasised the role of “emotion” and psychological factors in the outcome of physical stressors (Rose et al., 1979). Others, such as Sawrey, Conger, and Turrell (1956), argued rather that it was stress from psychical conflicts that were causal, and their experiments which posed behavioral conflicts between eating and punishment also showed increased ulcers in the conflicted rats. The research seemed promising. However, the only identified psychological factor that seemed to reliably hold up in the animal studies was the stress of being in an unpredictable, uncontrollable environment (Weiss, 1968, 1971; Murison & Overmier, 1990; Overmier, Murison, Skoglund, & Ursin, 1985); both unpredictability and uncontrollability seem the opposite of what characterises the situation of executives and managers, and would seem to be more applicable to lower socioeconomic status (SES) jobs. During this same period, physiologists and physicians established that either intermittent feeding that induced extended running in an exercise wheel over many days (e.g. Vincent, Paré, Isom, & Reeves, 1977 ) or hours of substantial physical restraint would produce ulcers in rats (Rossi, Bonfils, Liefoogh, & Lambling, 1956). Although the running model may be linked to some human exercise or labor situations, physical restraint as the main causal stressor is hard to link to the human situation, and yet it continues to be a popular model (Bonfils, 1993).

In the 1980s, Warren identified a bacterium in the stomachs of many humans, what we now call *Helicobacter pylori* (Hp). Marshall, working with Warren, established that a majority (58% to 100%, depending on the sample across a series of studies) of his patients presenting with peptic ulcer were infected with Hp (Marshall & Warren, 1984). They later showed that ingesting Hp resulted in gastritis and gastric inflammation. It was Marshall's argument that Hp was the primary cause of stomach ulcers—an argument initially not readily accepted in the medical community, in part because “everyone knew” ulcers were caused by stress. The importance of this discovery was recognised with the award of the Nobel Prize in Medicine to Marshall and Warren in 2005. Their discovery and its consequent publicity transformed the attitude of the medical community. Now ulcers were an infectious disease. The head of the US National Institutes of Health wrote an article virtually dismissing psychological factors and emphasising that Hp is directly causally responsible for ulcers (Hyman, 1994). This contemporary view that Hp is the main direct cause of ulcer disease (e.g. the Helicobacter Foundation, 2006) has led today to the near universal adoption of antibiotic therapy for ulcers as the essential treatment for peptic ulcer: “Kill the bug, cure the disease!” Psychology was suddenly seen as irrelevant. But is it? After all, brain-gut-brain interactive influences are well established—especially in the area of irritable bowel syndrome (see Enck, Martens, & Klosterhalfen, 2007; Gaman & Kuo, 2008; Mayer, 2011; Overmier, Murison, & Milde, 2006). These influences are mediated by several routes—nervous, endocrine, and immune.

The view that Hp is the main causal factor for peptic ulcer led to the adoption of antibiotic therapy to clear the stomach of Hp as the therapy of choice. This does indeed produce large percentages of successful patient outcomes, especially in those who have had symptoms for many months or years. But it is fair to note that the cure is neither ubiquitous nor necessarily permanent (Tovey & Hobsley, 1999). In addition, not all presenting ulcer patients are infected with Hp, particularly those that have had symptoms for 6 months or less (Boulos et al., 2002). Furthermore, some have reported that the rates of infection in patients with bleeding ulcer may be lower than in than in those with “uncomplicated” ulcers (54% vs. 65%; Tang et al., 2009), and in neither case was the rate of Hp infection close to 100 per cent.

Substantial percentages of the world populations carry Hp in their stomach. Comparisons across epidemiological studies are a challenge because the *base rates* of infections with Hp vary widely across the regions of the world—and even within countries—conflated with living conditions, and they change with time. There seems to be no correlation between general prevalence of Hp infection and prevalence of peptic ulcer (Hobsley, Tovey, & Holton, 2006). For example, in Korea the base rate of infection in adults over 17 fell from 78 per cent in the 1940s to 40 per cent in the 2000s (Do et al., 2009), but there has not been a similar decline in peptic ulcer disease (Tan & Goh, 2008), although one might speculate that changes in use of non-steroidal anti-inflammatory drugs (NSAIDS) might account for some of this stability.

## SCIENCE AND CAUSALITY

Whatever the merits of this antibiotic therapy viewpoint for the medical practitioner, inferring causality from the probability that presenting ulcer patients show infection [i.e. conditional probability of Hp given ulcer, Pr(Hp|U)] is, as science, weak. Such conditioning on the consequent (ulcer status) and then looking at the presumed antecedent (Hp infection)—a *retrospective* conditional probability—will always overestimate the association between the two (see Dawes, 1993). Because we are interested in *causality*, we are *not* concerned fundamentally with that *retrospective* relationship of percentage of ulcer patients that are positive for Hp (unless it is low). What *is* of interest to us is the *prospective relationship*: We want to know the conditional probability of ulcer given infection [Pr(U|Hp)], because Hp is the putative causal factor. These epidemiological data are hard to find because few studies sample the *general population* and *then determine ulcer status* (one positive example is Gasbarrini et al., 1995, discussed below). Of equal interest is the conditional probability of ulcer absent Hp [Pr(U|noHp)] and the conditional probability of no ulcer given Hp [Pr(noU|Hp)].

Although the conditional probability of ulcer disease given Hp infection is routinely higher than if there is no Hp infection, one cannot be certain which came first, the ulcer or the Hp infection. The probability of ulcer in the absence of Hp infection is reportedly between 5 and 20 per cent (Choung & Talley, 2008) or even more (Ciociola, McSorley, Turner, Sykes, & Palmer, 1999); some suggest that in countries with low Hp infection base rates and early access to physicians, 35–45 per cent of patients with duodenal ulcers are Hp negative (e.g. Tang et al., 2009). It may be that the presence of gastrointestinal erosions provides the breeding ground for chronic Hp infection. Indeed, we have already noted that early cases of peptic ulcer are less likely to be accompanied by Hp infection than are chronic cases, while in contrast, if study is restricted to patients already with bleeding duodenal ulcers, infection may be found in up to 100 per cent (Gisbert et al., 2001).

However, the most striking fact to be gleaned from the epidemiological studies of the general population is that the vast majority of Hp infected individuals are asymptomatic. We can see this in an epidemiological study (Gasbarrini et al., 1995) of the general population of San Marino, which found a base rate of about 50 per cent Hp infection (based on serology), higher than some countries but lower than in others. From their report we can calculate the conditional probabilities that guided Marshall and Warren, as well as those conditional probabilities that are of more interest to those seeking to understand causality. Gasbarrini et al. found that the *retrospective* conditional probability of Hp infection given that there was ulcer was 0.70 [Pr(Hp|U)]. But Gasbarrini et al.'s *prospective* conditional probabilities suggest a rather different picture: The conditional probability of ulcer given Hp infection was 0.18 [Pr(U|Hp)], while the conditional probability of ulcer given no infection was lower at 0.08 [Pr(U|noHp)], but not insignificant. Nearly a third of the people with ulcers did not or never had harbored Hp. But the most striking datum is that the conditional probability of being ulcer-free given that they had infection with Hp was .82 [Pr(noU|Hp)]. While it was clear that infected persons were more than twice as likely to have an ulcer as uninfected persons, infected persons were more than four times as likely to *not* have ulcer despite being infected. These relations and their values are shown in Table 1. These are not consistent with the view that Hp infection is the simple direct primary direct causal factor for ulcer disease (Marshall, 2006; see also the Helicobacter Foundation, 2006).

**TABLE 1:** Prospective Conditional Probabilities of Ulcer or Not as a Function of Helicobacter Pylori Infection or Not (Computed from Data of Gasbarrini et al., 1995)

		*Current or Prior Infection with Helicobacter Pylori*	
		
		*No*	*Yes*
Presenting with ulcer	Yes	P (U| no *Hp*) = 0.08	P (U|*Hp*) = .18
	No	P (no Ulcer| no *Hp*) = 0.92	P (no Ulcer|*Hp*) = .82

What we can infer from this overview is that, at best, Hp clearly can contribute to the severity and persistence of ulcer disease, but that Hp cannot be the single simple causal factor. Hp may infest an ulcer rather than being causal. Vulnerability to ulcer and thence infection of those ulcers by Hp must have other antecedents. That is, Hp may be at best a co-factor in ulcer processes when and if other permissive or causal factors are present. Indeed, some now even refer to Hp as a “secondary complication” (Tovey & Hobsley, 1999) that may slow or prevent ulcer healing, or even contribute to increased severity of an existing ulcer. Consistent with this approach, preclinical data have shown that bacteria rapidly colonise and modulate the healing of gastric ulcers (Elliott, Buret, McKnight, Miller, & Wallace, 1998).

Intriguingly, dose-dependent healing (up to 44%) of duodenal ulcer through “treatment” with placebo has been reported, suggesting the therapeutic importance for the ulcer patient of consultation and of coping actions such as taking a course of—albeit placebo—treatments (de Craen et al., 1999); such placebo effects implicate the importance of “psychology” in peptic ulcer.

## CORRELATES OF ULCER DISEASE

Although there is a correlation between ulcer disease and Hp infection, the direction of the causal arrow is certainly not clear. Perhaps we need to consider other possible causes of ulcer. A number of correlates of ulcer have been identified in epidemiological studies. There is a substantial literature showing a prospective association between life activities and stresses and the vulnerability, persistence, or recurrence of ulcers (e.g. Ellard, Beaurepaire, Jones, Piper, & Tennant, 1990) even after Hp eradication (see Levenstein, 2002, for a review). Identified correlates to ulcer include Hp infection, smoking, heavy alcohol consumption, physical workload, socioeconomic status, hospitalisation in intensive care and post-surgical care, and life stresses. Levenstein, Kaplan, and Smith (1997) provide an epidemiological overview of these co-factors. Let us briefly consider a sample of these.

### Non-Steroidal Anti-Inflammatory Drugs (NSAIDs)

The use of aspirin or other NSAIDs as pain-relieving drugs requires first consideration because data indicate that it is indeed a critical co-factor, and increasingly so as the use of NSAIDs increases worldwide. NSAIDs are commonly recommended to aging persons as protective against heart infarcts, brain plaque accumulation and cognitive decline, or relief from arthritis, gout, and other chronic pain conditions. NSAID use is substantially associated with gastric bleeding (Gisbert et al., 2001; Weil et al., 1995). NSAID use and resulting direct mucosal damage may provide the possibility for chronic impairment of stomach integrity and for opportunistic infection by Hp.

However, most of the everyday use of NSAIDs is not prescribed by physicians and is used to provide relief from headaches or pains that have as their causes experiential stresses. To the extent that this is so, NSAID use as a co-factor in ulcer may well often be at root a psychology problem. For example, Brady and Fiero (2001) state that “primary headaches are most frequent (90%) and cannot be explained by brain abnormalities … or medical disease”. Only 10 per cent have identifiable medical causes. More than 68 per cent of Americans report episodes of “tension” headaches, and many of these will self-medicate with NSAIDS. In sum, a not insignificant part of NSAID use—and associated ulcer—is secondary to challenges arising in the experiential life-space of individuals with inadequate coping behaviors. Thus, the chronic use of NSAIDs is not easily identified as an independent sole causal factor in ulcer.

### Smoking and Nicotine

Cigarette smoking has long been regarded as an important contributor to ulcer disease. Nicotine, while without effect itself, is known to sensitise the stomach to pentagastrin, which stimulates acid secretion (Robert, Stowe, & Nezamis, 1971). Parasher and Eastwood (2000) report that smokers are more likely to develop ulcers, that these are more difficult to heal, and that relapse of ulcer disease is more likely in smokers. Smokers seem to be at higher risk of becoming infected with Hp which likely plays a role in these observations.

But again, as psychologists, we ask “Why do people smoke?” There are of course many different reasons. Youths often do so because of social anxiety reasons such as to “fit in”, to appear more mature, and to rebel against social constraints. Jacobs (1997) argues, “Adults smoke for other reasons. They may have a lot of stress and pressures because of economic and personal problems. They may be unemployed or working but not making enough money to take care of themselves and their families. They may be homeless, or they may be dealing with alcohol or cocaine/heroin addictions. Some may be in bad marriages or relationships in which there is physical and/or verbal abuse. All these people may smoke to feel relaxed or to give them energy while going through a hard time.” Others do so to control their appetites and weight, while still others say they do so for relaxation and pleasure. While nicotine is actually a stimulant, it does release dopamine in the brain, which is commonly associated with feelings of reward. None of these reasons are medically based but are psychological, some of which are linked to experienced tensions and anxieties, many of which are more likely for those in low SES groups. In sum, a not insignificant part of smoking may be secondary to challenges arising in the behavioral and experiential life-space of individuals with inadequate coping behaviors (Jarvis, 2004).

### Alcohol Consumption

Very heavy alcohol use has been associated by some with peptic ulcer and mortality from ulcer (e.g. Razvodovsky, 2006). On the other hand, surveys that include more moderate drinking (Chou, 1994) have found little linkage between alcohol consumption and ulcers. Whether this is because heavy drinking ultimately impairs liver function or because moderate alcohol drinking actually protects against Hp is unknown. Nonetheless, prospective epidemiological studies continue to identify alcohol as a risk factor (Levenstein et al., 1997).

Again, we need to ask about the reasons for heavy alcohol consumption. One commonly hears the phrase “Drowning one's sorrows in drink”, suggesting that heavy alcohol consumption is a form of self-medication (see Robinson, Sareen, Cox, & Bolton, 2011). Thus, alcohol drinking may result from poor work environment conditions, poor marital relations (avoidance of going home), or poor social relations (Marchand, Parent-Lamarche, & Blanc, 2011)—or even be causal of bad social relations.

### Work Conditions

Heavy labor is associated with peptic ulcer (Sonnenberg, 1988). This is consistent with preclinical findings that extreme physical exercise influences stomach function and can result in gastric ulcerations (Vincent et al., 1977). Occupational stresses have been linked to ulcer-like symptoms—especially in those that have poor active coping behaviors or lack good social supports (Chen, Wong, & Yu, 2009). In addition, labor under shift-work schedules is also associated with increased peptic ulcer (Pietroiusti et al., 2006). Although more physically demanding, heavy labor is usually less well paid. Heavy labor under conditions of uncertainty of employment is more common for those in the lower SES with its associated factors.

### Socioeconomic Status (SES)

Ulcers are more common in low socioeconomic groups (LeClere, Moss, Everhart, & Roth, 1992). Lower SES is associated with higher base rates of Hp infection, although as we have noted above, such base rates do not reliably correlate with development of ulcer, but only with severity. That SES shows up as a relevant factor is not surprising because hygiene, smoking, heavy alcohol use, and heavy physical labor are also correlates of low SES and account for much of the SES component (Levenstein & Kaplan, 1998). But in addition, low SES carries with it its own special burdens and tensions, often related to unavailability of affordable housing, inadequate food for families, and risk of dismissal from employment. Furthermore, income insecurity is linked to marital insecurity and disrupted sleep patterns. All of these factors contribute to allostatic load (McEwen & Lasley, 2007) which constitutes a risk factor for ulcer (see Sapolsky, 2004, for a fascinating discussion).

### Stress

Stressors can include physical ones (e.g. trauma, pain, etc.) or psychological ones (e.g. fear, anxiety, insecurity, bad social relations) and are characterised by unpredictability and uncontrollability (Koolhaas et al., 2011; Mineka & Kihlstrom, 1978). All stressors activate the hypothalamic-pituitary-adrenal-cortex axis (HPA axis) and commonly the sympathetic-adrenal-medullary axis. Each has significant effects in the gastrointestinal tract. These influences range over mast cells in the stomach and histamine production (which are known to be subject to classical conditioning; MacQueen, Marshall, Perdue, Siegel, & Bienenstock, 1989), acid production, mucosal circulation and immunity, gastric motility and emptying rate, and mucosal integrity (Bhatia & Tandon, 2005). Acid is a primary proximal aggressive agent in the production of stomach ulcer (Weiner, 1991), while circulation and motility are linked to mucosal resistance to acid. And, as noted above, Wolff and Wolf showed that acid production in the stomach is modulated by emotional state. More recently, both clinical and preclinical studies have shown that psychological stress enhances Hp infection and colonisation of the stomach and that this is facilitated in part through glucocorticoids (Graham & Osato, 2000; Guo et al., 2009, respectively). Mongolian gerbils and mice infected with Hp *and* subjected to the stress of restraint in water develop more gastric damage than uninfected animals, the unstressed animals showing no pathology (Yamamoto et al., 2000). Thus, there should be no question that the psychological status of the organism is linked to gastric health, in line with a stress-diathesis model of disease (Monroe & Simons, 1991). The psychological status, directly through forms of stress and its products, or indirectly through bad habits or self-medication, may make gastric mucosa more vulnerable to erosions and Hp infection.

## ABANDONING THE SEARCH FOR PRIMARY CAUSALITY

The focus now is how psychological factors contribute to ulcer disease. Certainly, the epidemiological research on correlates of peptic ulcer reviewed above suggests possible causal factors. What has always been sought is experimental evidence that psychological factors matter. Historically, the initial efforts by psychologists, typified by research in the mid-20th century, were attempts to empirically demonstrate the *precipitating* power of psychological processes—attempts to demonstrate *primary direct* causality using psychological-behavioral methods, typically in animal experiments. Animal model experiments were (and are) used to test empirically for causality because of the ethical constraints against experimentally inducing debilitating disease in humans. In these, animals might be placed in a situation involving psychological conflict (e.g. being punished for eating) to determine if this “conflict” induced ulcers. Or, they might be put in situations requiring that they work to prevent irregular aversive events for themselves and others. To make a short story shorter, sometimes gastric ulcers were observed, but commonly they could be attributed to unintended causes. Careful analyses and follow-up studies showed that many of these results were more likely attributable to experimental design error or self-starvation (see Glavin et al., 1991). For the most part, experiments of this sort were not successful in establishing psychological-behavioral manipulations as *primary*, *direct*, *causal* factors of ulcer. And as well, we have seen that the currently claimed primary causal status of Hp is also suspect, although its role in exacerbation of ulcers is likely certain.

## A NEW EMPIRICAL APPROACH: SEARCH FOR MODULATION

In our own preclinical research, we adopted a different strategy. We sought to show that psychological factors can *modulate vulnerability to gastric ulcers produced through a different ulcer-inducing challenge*. Our goal has been to show that life events and how they are *perceived* also changed the degree of stress and thus modulated (a) vulnerability to ulcers, (b) the precipitation of ulcers, and (c) the persistence of ulcers.

**FIGURE 1:**
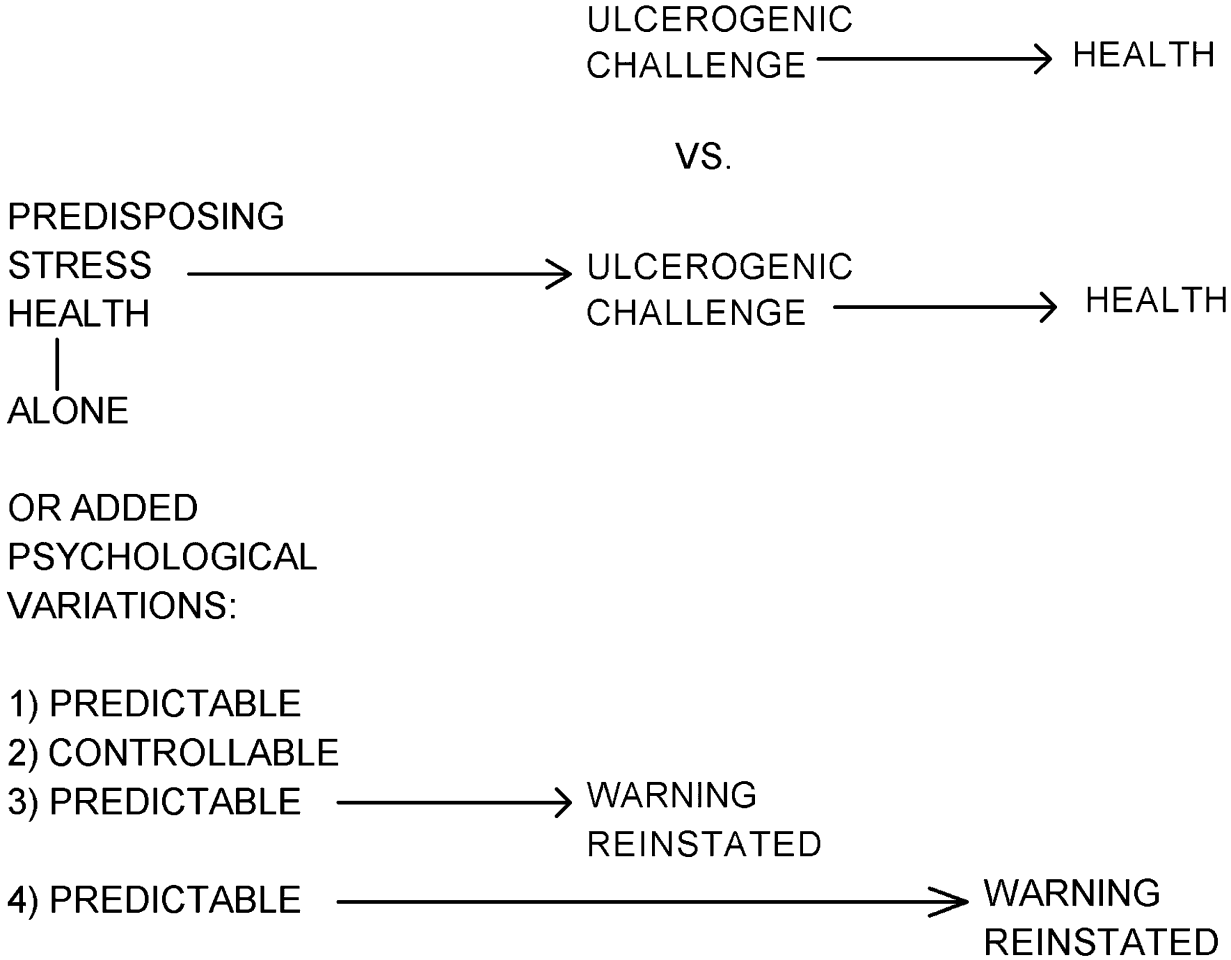
Flow chart of our experimental strategy.

To study psychological factors as modulatory factors in the ulcer process, you need something to modulate. We used an ulcer-inducing challenge—mild restraint in room temperature water (RiW)—a psycho-physical event that by itself induces a significant amount of gastric ulcer-like erosions. We thought that our method (Overmier, Murison, & Ursin, 1986), rooted in our reading of Richter's work (1957) on parasympathetic activation in rats subjected to a swimming task, was a novel one, but later learned that Japanese colleagues had developed a similar effective ulcer-induction technique (e.g. Osumi, Takaori, & Fujiwara, 1973). We showed that these ulcerations are dependent upon the animal being conscious (Murison & Overmier, 1993). Then, to demonstrate modulation of this basic ulcerative process, we added other manipulations. This strategy is sketched in Figure 1.

**FIGURE 2:**
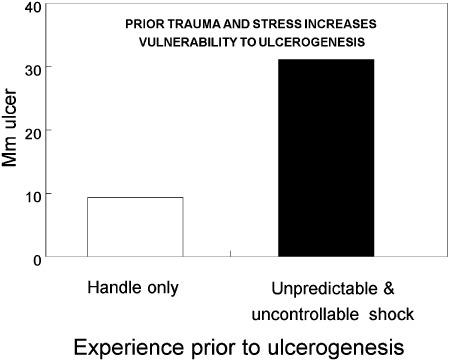
Increased vulnerability to ulceration as a consequence of prior trauma.

We first asked whether having had a prior stressful experience changed the vulnerability of the animals to the RiW ulceration. To do this we exposed experimental rats to the potentially predisposing stressor, allowed them to recover, and then days (or even months) later exposed them to the RiW challenge. The prior stress experience we used was exposure to a relatively short series of uncontrollable and unpredictable aversive events (brief electric footshocks); Weiss (1971) had previously shown that such treatment when very intense and prolonged was itself ulcerogenic. We knew that the shorter, milder treatment that we used did not itself induce gastric erosions (Overmier & Murison, 1989) but in pure psychological-behavioral experiments resulted in later psychological and behavioral debilitations (e.g. Maier, Coon, McDaniel, Jackson, & Grau, 1979; Overmier & Seligman, 1967) that have been linked to learned helplessness, neuroses, and depression. We found that an initial exposure to such events also resulted in significant increases in later RiW-induced gastric ulceration (e.g. Overmier & Murison, 1991). This effect of prior stress on later vulnerability is illustrated in Figure 2,^1^ and it is thought to mediate its effects through opioid-related mechanisms (Overmier & Murison, 1994).

Our subsequent experiments were designed to show whether *psychologically important variations* of the context of prior stressful experiences modulated the degree of exacerbated vulnerability to the ulcerogenic challenge (see Figure 1).

But what do we mean by “psychologically important”? Evolved organism-environment “coping” capacities include both the psychological and the behavioral. The capacity to anticipate environmental events allows the organism to react anticipatorily (e.g. classical conditioning). Also, there is the capacity to act on—to control—the environment and to learn which actions are beneficial (e.g. instrumental learning). We know from learned helplessness experiments that failures of or absence of these coping processes result in psychological, behavioral, and physiological consequences for modern mammals (see Overmier & LoLordo, 1998, for a review). Thus, we chose to introduce these psychologically important features of predictability and of controllability of the predisposing stressors to assess whether these influenced the vulnerability to the ulcerogenic challenge of RiW.

### Amelioration of Vulnerability by Prediction

**FIGURE 3:**
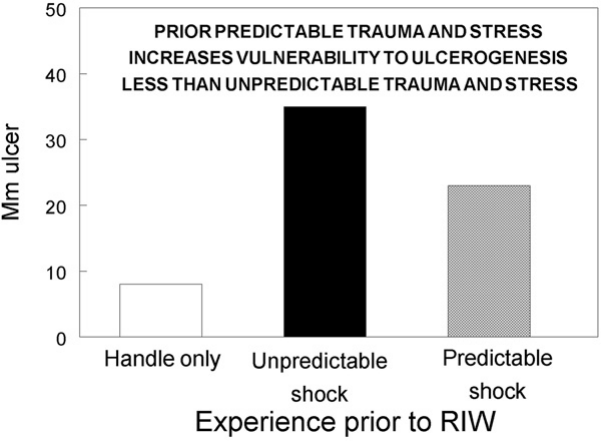
Modulation of effect of prior trauma effect by predictability.

Although exposure to prior stressors may typically increase vulnerability to ulcer, we now want to assess whether this increased vulnerability is inevitable. Predictability of events is a relationship known to be psychologically important. Relevant here is a comparison between two groups that receive an identical series of initial stressful events, but one group receives a short warning signal before each event, while the other group receives them unsignaled. The results of such a comparison are illustrated in Figure 3.

We found that providing prediction of these initial aversive events reduced later exacerbation in ulcer (Overmier et al., 1985). That is, the predictability of the prior stressor reduced the typical stress-induced vulnerability to the induction of gastric ulcers.

### Amelioration of Vulnerability by Behavioral Control

**FIGURE 4:**
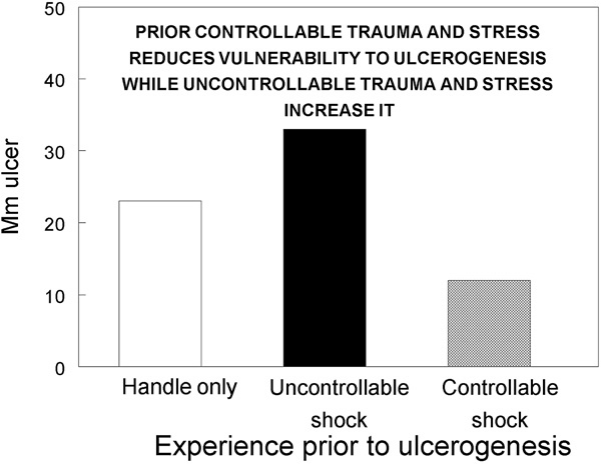
Modulation of effect of prior trauma by controllability.

We also assessed whether behavioral control of events will alter the degree to which prior stressful events increase vulnerability to gastric ulcers. Relevant here is a comparison between two groups that receive an identical series of initial stressful events, but one group can control the event's duration because it is possible to escape from each stressor, while the other group has no control. The two groups are linked such that the durations of the individual events are matched (yoked) between the two groups. Both groups then receive RiW challenge and can be compared as well to a group that experiences restraint-induced ulcers without any such prior stress experience. The results of such a comparison are illustrated in Figure 4.

We found that providing behavioral control over these aversive events reduced the proactive exacerbation of gastric ulceration produced by equal uncontrollable shocks (Murison & Isaksen, 1982). In fact, these experiences of acquiring mastery of the environment through control *reduced* the degree of vulnerability to gastric ulceration to a level *below* that of another comparison group that received no prior stress experience.

These three demonstrations illustrate that while experiences with prior stress *can* increase vulnerability to gastric ulceration, the degree of vulnerability is modulated by whether or not the prior stressors are (a) unpredictable and uncontrollable, (b) predictable, or (c) controllable. In fact we saw that learning to control stressors may even be *protective* against future ulcer. In sum, it is not the prior stress events themselves that are critical, but the *psychological context* in which they occur that influences future vulnerability.

### Exaggeration of Ulcerogenesis by Threat

We saw above that providing prediction of stressors reduced later vulnerability to RiW-induced ulcers. This was in one sense expected, but in another it was surprising because signals for aversive events have been demonstrated to be themselves aversive (Overmier & Leaf, 1965). That is, presentations of signals that have previously signaled aversive events elicit “fear” and “anxiety”. Similarly, environments and contexts in which organisms have had prior traumatic experiences are threatening as well, and elicit anxiety (Fanselow, 1980). Can the presentation of such important threat stimuli during an ongoing ulcerogenic process exacerbate the degree of induced ulceration?

**FIGURE 5:**
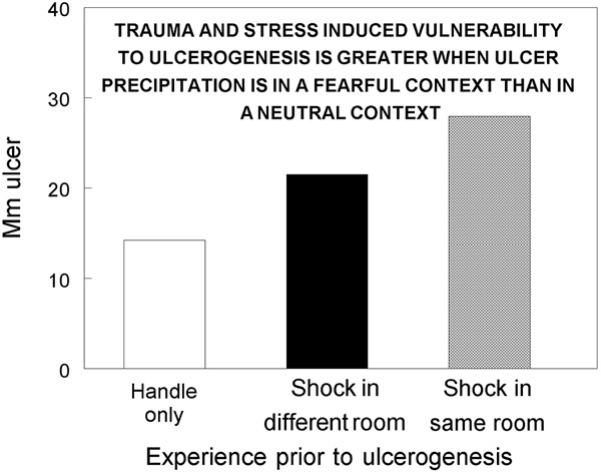
Exacerbation of effect of prior trauma by exposure to a fearful context during ulcer-induction.

To assess this we compared groups both of which had had experience with prior signaled shocks in a given environmental context. For one group the RiW-ulcer-induction took place in that shock-associated environment; that is, the ulcer induction process took place in a context that was threatening to the organism. For another group, the RiW-induction took place in a different, non-threatening environmental context. Figure 5 shows that threat during ulcer induction exacerbated the degree of ulceration (Murison & Overmier, 1990).

### Augmented Persistence of Ulceration by Threat

**FIGURE 6:**
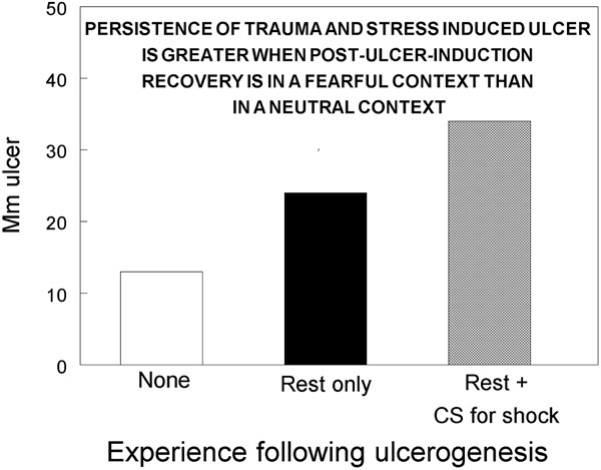
Exacerbation of effect of prior trauma by exposure to a fearful context during recovery following ulcer-induction.

Finally, we assessed whether experiencing psychologically threatening events *after* the ulcer induction could increase ulcer expression. For this experiment we drew upon the fact noted above that signals for aversive events are themselves aversive. Here the primary contrast is between two groups, both of which have previously experienced prior stress experience of signaled shock events, and then later experienced the RiW-induction of ulcers. Consequently, the animals were allowed to rest for a couple of hours. During this rest period, one of the two groups experienced repeated presentations of the previously established warning signal; the other pre-stressed group heard no such signals. The animals experiencing psychologically threatening events *after* the RiW-ulcer-induction displayed increased ulcer relative to those that had not heard such threats (Overmier & Murison, 1989). This is illustrated in Figure 6.

### Summary of Results from New Strategic Approach

In summary, we have demonstrated in preclinical studies that when an organism is undergoing stresses—stresses that will increase its later vulnerability to physical dysfunction and disease—the availability of effective *psychological-behavioral* coping strategies reduces the organism's later vulnerability even though the total physical aversive events are matched physically. Moreover, the presence of psychological threat during the ulcer induction process increases the extent of gastric ulceration. Finally, the presence of threat after the ulcer induction process also influences the degree of ulceration either by exacerbation of the prior ulcers or by increasing the persistence of the induced ulcers, or both. We believe that these results provide an *experimental* foundation for assuming a significant role for psychological factors in predisposing, modulating of precipitation, and sustaining of gastric ulceration in humans.

The purpose of our strategic approach and model was to show that psychological-behavioral variables have important proactive effects that *modulate* organismic vulnerability, ulcerogenesis, and persistence. We believe our data combined with observations of others make it clear that ulcer disease is *not* best understood *only* in terms of local processes but rather in terms of the extended experiential and psychological history of the organism.

## IMPLICATIONS FOR TREATMENT OF PEPTIC ULCER

Our work challenges those who want to disregard the relevance of psychological processes, in favor of either an exclusive focus on bacteria or on bacteria plus NSAIDs. While we are not advocating a return to psychoanalytic treatment, the reviewed experiments on modulation challenge all to look at the total functioning person in his or her social environment—actual successes and failures in life (behavioral, familial, social, economic, and cognitive), experienced stresses (assaults, war, terrorism, and cataclysms), and perceptions of them—to identify the operative socio-behavioral factors that *taken together* with local biological factors cause and sustain gastric ulcer disease.

Every single patient likely needs assessment for need for remediation of (a) Hp infection, (b) behavioral factors such as smoking and drinking, (c) social relationships, (d) social-environmental (SES) factors, and, last but not least, (e) past and current stresses and the coping ability of the individual. Peptic ulcer is truly an integrated organismic response; it is an interdependent play of organismic consequences spanning behavior, emotions, anticipations, as well as physiological processes in brain and gut. While Hp is not irrelevant, it—even coupled with other stomach-injuring consumables—is not the final single answer Hyman implied (Hyman, 1994). Certainly, Hp eradication is called for in cases of long-standing or persisting peptic ulcer. In addition, cognitive and behavior therapies addressing smoking and alcohol misuse, social behavior, family challenges associated with sleep and diet, coping with job and economic problems, as well as work with employers on workload and schedule management are all part of the counseling and treatment protocols that need to be implemented to assure a cure for peptic ulcer. Physiological reductionism alone simply will not do.

The role for psychologists (and other social scientists) on the health team is clear. Most of the relevant co-factors noted require psychological assessment and psychological behavioral management. Some factors require medical management (e.g. acid production and Hp elimination). The deep dualistic-based rift between medicine and psychology should be replaced with a biopsychosocial model that structures the spectrum of treatments to be applied for gastric ulcer (Levenstein, 1998; Novack et al., 2007).

Ulcer disease will only be truly curable when we stop seeking single dimension causes in terms of mind *or* body. Only when we together fully reject a simplistic Dualism can both psychology and medicine—as cooperative health professions—flourish and find effective and efficient cures for peptic ulcer, as well as other disorders labeled as psychosomatic disease. Let us—psychologists and physicians—now move on and dance together in a celebration of advancing human health and well-being.

## Footnotes

1 Although in this article we show only the results from the critical comparison groups, in fact all our experiments include several other control and reference groups. The reader is directed to them for the fuller scientific comparisons.
